# The difference of the retinal structural and microvascular characteristics in patients with MOGAD-ON and AQP4-ON

**DOI:** 10.1186/s12883-022-02848-2

**Published:** 2022-08-27

**Authors:** Yajun Yao, Xindi Li, Yun Xu, Xiaofang Liang, Liu Yang, Fu-Dong Shi, Xinghu Zhang, De-Cai Tian, Xuxiang Zhang

**Affiliations:** 1 Department of Neurology, Beijing Tiantan Hospital, Capital Medical University, Beijing, 100070 China; 2 Department of Ophthalmology, Beijing Tiantan Hospital, Capital Medical University, Beijing, 100070 China; 3 China National Clinical Research Center for Neurological Diseases, Beijing, 100070 China; 4 Department of Neurology, Tianjin Neurological Institute, Tianjin Medical University General Hospital, Tianjin, 300052 China

**Keywords:** Optic neuritis, MOGAD, NMOSD, OCTA

## Abstract

**Background:**

Antibodies against myelin-oligodendrocyte-glycoprotein (MOG-Abs) associated disease (MOGAD) has been recognized as a disease entity. Optic neuritis (ON) is the most common symptom in MOGAD. To demonstrate the differences in retinal microvascular characteristics between patients with MOGAD-ON and aquaporin-4 antibody (AQP4-Ab) positive ON.

**Methods:**

In a prospective study, optical coherence tomography (OCT) and optical coherence tomography angiography (OCTA) were used to measure retinal and microvascular parameters.

**Results:**

Twenty-six MOGAD-ON eyes, 40 AQP4-ON eyes, and 60 control eyes were included in the study. The thickness of RNFL and GCC in MOGAD-ON eyes was significantly lower than that of HC (*p* < 0.001, respectively), but comparable to AQP4-ON eyes. The vessel density in retina capillary plexus (RCP) was reduced significantly in MOGAD-ON than that in AQP4-ON (*p* < 0.05, respectively). The visual accuracy was positively correlated with vessel density of superficial RCP in MOG-ON (*p* = 0.001) and positively correlated with the thickness of the inner retina layer in AQP4-ON (*p* < 0.001).

**Conclusion:**

The retinal neuro-axonal damages between MOGAD-ON and AQP4-ON were comparable. Unlike AQP4-ON eyes, microvascular densities were significantly reduced in MOGAD-ON and were positively correlated with the deterioration of visual acuity in MOGAD-ON.

**Trial registration:**

Clinical and Imaging Patterns of Neuroinflammation Diseases in China (CLUE, NCT: 04106830).

## Introduction

Antibodies against myelin-oligodendrocyte-glycoprotein (MOG-Abs) have been detected in several autoimmune central nerve systems (CNS) disorders, in which the clinical characteristics often overlap with aquaporin-4 (AQP4) Ab-negative neuromyelitis optica spectrum disease (NMOSD) [1, 2]. Recently, MOG-Ab associated disease (MOGAD) has been recognized as a disease entity in their own right due to the immunopathology mediated by MOG-Abs [1, 3, 4]. Optic neuritis (ON) is the most common neuro-ophthalmic symptom in MOGAD, accounting for approximately 78% of MOGAD patients [5, 6]. MOGAD-ON manifests as recurrent episodes and severe visual impairment (even blindness) with optic disc swelling and extensive optic nerve lesions [7]. However, unlike NMOSD-ON with poor prognosis [8, 9], the visual acuity is often preserved in MOGAD-ON because it responds well to steroids [10].

Both optical coherence tomography (OCT) and optical coherence tomography angiography (OCTA) are non-invasive imaging techniques used to visualize the microstructures of the fundus. OCT performs a real-time and in vivo retinal biopsy to directly visualize the microstructure of human eye tissue, including the peripapillary retinal nerve fiber layer (pRNFL) and ganglion cell complex (GCC) [11]. The pRNFL comprises axons originating from ganglion cell neurons and reveals optic nerve status, and macular GCC is a direct reflection of the intrinsic ganglion cell bodies, which can provide information about primary retinal pathology [12]. OCTA generates high-resolution information on retinal and choroidal vessels, and gains a better visualization of vasculature around the optic disc and macula [13]. As cerebral and retinal vasculatures are anatomically interconnected with similar features, a retinal vascular study would shed light on the underlying pathogenesis of microvascular impairment and neuronal damage in the central nervous system.

With the utilization of retinal OCT in clinical neuroimmunology, researchers successively observed that pRNFL and GCC were significantly thinning in NMOSD-ON [14, 15] and MOGAD-ON [10, 16], and were consistent with their visual acuity loss. Similar to the peripapillary vascular attenuation reported in NMOSD-ON [10], the retinal vascular density also decreased in MOGAD-ON and was positively correlated with the number of ON episodes [17]. The evidences above indicated that the irreversible retinal damages were not only presented in NMOSD-ON, but also in MOGAD-ON. The retinal destructions in NMOSD primarily result from the irreversible injuries of AQP4-Abs to Müller cells in the retina [18]. But, in MOGAD-ON, the pathologies of afferent visual system damages mediated by MOG-Ab are less well understood. As studies to compare retinal microstructures and ocular vasculature in MOGAD-ON and AQP4-Abs-positive NMOSD-ON (AQP4-ON) are limited, the severity of retinal damages following MOG-Abs and AQP4-Abs associated ON have been inconclusive. In this study, we separately investigated the retinal structural and microvascular characteristics of patients with MOGAD-ON and AQP4-ON, to demonstrate the distinct underlying pathologies of visual impairments mediated by MOG-Abs and AQP4-Abs, which is essential for refining diagnoses and tailoring treatments.

## Materials and Methods

### Study participants

We performed a prospective study, Clinical and Imaging Patterns of Neuroinflammation Diseases in China (CLUE, NCT: 04106830). Patients with a history of ON were enrolled from Jan. 2019 to Jan. 2022 in the neurology department of Beijing Tiantan Hospital, Capital medical university. ON was defined as the presence of an acute relapse lasting more than 24 hours and associated with decreased high-contrast letter acuity, pain with eye movement, color desaturation and/or visual field (VF) abnormalities, with or without optic nerve swelling or enhancement visualized by magnetic resonance imaging (MRI). Ethical approval for this study was obtained from the Institutional Ethics Committee at Beijing Tiantan Hospital.

The inclusion criteria for patients were as follows: (1) best-corrected visual acuity (BCVA) of the eye with better eyesight ≥20/400, intraocular pressure (IOP) ≤ 21.0 mmHg, a spherical equivalent less than 3.00 D; (2) the duration more than 6 months since last ON attack [19]; (3) serum MOG-Ab or AQP4-Ab positive tested via cell-based assay (CBA); (4) able to perceive the light spot during OCT and OCT-A examinations and cooperate with the examiner. Eyes with concomitant potentially confounding diseases (glaucoma, diabetes mellitus, retinal surgery, retinal disease) were excluded. The inclusion and exclusion criteria of health controls (HCs) were similar to those for the MOGAD and NMOSD group except for the BCVA≥20/25, absence of eye diseases, and the absence of serum MOG-Ab and AQP4-Ab.

The AQP4-IgG antibody and MOG-IgG antibody testing were performed blindly at the Euroimmun Medical Diagnostic Laboratory (China) using a fixed-cell-based indirect immunofluorescence test on BIOCHIPs (EUROIMMUN AG, Lübeck, Germany). Information including demographic data, disease duration of ON, time since previous ON attack, ON relapse times and status of MOG-Abs and AQP4-Abs were collected and reviewed. The evaluation of EDSS was evaluated independently by two neurologists according to a predefined standard on the same day as the ophthalmoscopic examinations.

Early Treatment Diabetic Retinopathy Study (ETDRS) charts (Precision Vision, USA) were used to collect the prognostic Best-Corrected Visual Acuity (BCVA) of the patients with ON. The presented letter-acuity scores may be converted to LogMAR (logarithm of the minimum angle of resolution) as follows: LogMAR = −(0.02) * Letters + 1.1 [20]. Due to statistical availability, VA (logMAR) < 0.0 is regarded as 0.0, VA (logMAR) > 1.0 is regarded as 1.0, resulting in a range of VA (logMAR) from 0.0 (corrected decimal VA ≥ 20/20) to 1.0 (corrected Decimal system) VA ≤ 20/200) [21]. The intraocular pressure was measured by Canon TX-20 full auto non-contact tonometer (Canon Inc., Tokyo, Japan).

### OCT

The thickness measurements of pRNFL, inner retina, and outer retina were performed with an RTVue-XR Avanti spectral-domain OCT (Optovue, Inc., Fremont, CA, USA; software version 2017.1.0.155). Retinal imaging was firstly performed by a three-dimensional (3D) reference scan, which was used as the reference and registration image for the enhanced macular map 5. It consisted of a dense grid scan in a 6 × 6 mm central macula area. The optic nerve head (ONH) scan consisted of 13 concentric rings with diameters ranging from 1.3–4.9 mm and 12 radial lines with 3.4 mm in length. The thickness of the retinal nerve fiber layer (RNFL) was measured at a diameter of 3.45 mm around the center of the disk using the ONH protocol. RNFL analysis was done in 8 sectors namely superotemporal (ST), superonasal (SN), inferotemporal (IT), inferonasal (IN), nasal inferior (NI), nasal superior (NS), temporal inferior (TI), and temporal superior (TS).

The perifoveal retina was divided into the inner retina and outer retina. The perifoveal retina scan is 1 mm temporal from the foveal center, and consists of 15 vertical line scans, covering a 7 mm square area [22]. The ganglion cell complex (GCC) was referred to the inner retina which comprised the RNFL, the ganglion cell layer (GCL), and the inner plexiform layer (IPL). The outer retina was measured from the inner plexiform layer (IPL) to retinal pigment epithelium (RPE) layer, which consisted of the inner nuclear layer (INL), outer plexiform layer (OPL), outer nuclear layer and inner segment layer (ONL + ISL), outer segment layer (OSL) and retinal pigment epithelium (RPE).

### OCTA

OCTA scans of the optic disc (4.5 * 4.5 mm) and macula (6 * 6 mm) were obtained by a spectral domain system (RTVue-XR Avanti, Optovue, Fremont). The radial peripapillary capillary network was visualized on scans within a 1.0 mm wide elliptical annular region extending outward from the optic disc boundary, and the vasculature within the internal limiting membrane and the nerve fiber layer were analyzed automatically using the software.

The parafoveal capillary network was observed on scans of a circular area with a diameter of 1 mm–3 mm surrounding the fovea, while the scan of the 3 mm–6 mm annular area around the parafoveal capillaries was a perifoveal capillary network. Both parafoveal and perifoveal capillary networks were further divided into superior and inferior hemispheres, temporal section (TEM), nasal section (NAS), inferior section (INF) and superior section (SUP). The superficial retinal capillary plexus (SRCP) was analyzed from 3 mm below the internal limiting membrane to the outer boundary of the IPL, and the deep retinal capillary plexus (DRCP) was referred to the layer from IPL to ONL. Macular layer segmentation was automatically recognized by the software inside the machine (Optovue, Inc., Fremont, CA, USA; software version 2017.100.0.1). The vessel density was calculated as the percentage area occupied by the large vessels and micro-vessels in the analyzed region. They were automatically generated in the whole scan area and all sections using the software (V.2017.100.0.1, Optovue, USA). Two experienced ophthalmologists blindly evaluated the quality of OCTA images to determine interobserver reproducibility (Kappa = 0.834, *p* < 0.001). Poor-quality photographs with a signal strength index less than 40 or images with residual motion artifacts were rejected. In terms of the OCT and OCTA acquisition, we followed OSCAR-IB [23, 24]and APOSTEL [25] criteria to assure enough good quality of the images.

### Statistical Analysis

SPSS Statistics version 22 (IBM, Armonk, NY), and GraphPad Prism version 7.0 (GraphPad Software, La Jolla, CA) were used to analyze and create graphs. Data were presented by n (%), mean (SD) or median (range). The t-test or Mann–Whitney test were used to compare demographic characteristics and clinical presentations. The chi-square test was used to compare the proportion of eyes with reduced RNFL in each quadrant. The generalized estimating equation (GEE) was used throughout the analysis whenever applicable for adjusting for age, gender, disease duration and the inter-eye correlation. Univariate and multivariate linear regression methods were used to analyze the effects of OCT and OCTA parameters on visual function. We considered outcomes with a *p* value of < 0.05 at univariate analysis as candidates for multivariate regression modeling. Collinearity and interaction between variables were checked before regression analysis. A *p* < 0.05 was considered statistically significant.

## Results

### Demographic and clinical characteristics of the MOGAD-ON and AQP4-ON

Seventeen MOGAD-ON (26 eyes), 26 AQP4-ON (40 eyes), and 30 HC (60 eyes) participants were included in the study. The demographic and clinical features of patients and healthy controls were presented in Table 1. MOGAD-ON occurred at a similar age as AQP4-ON. The proportion of females in the MOG-ON (52.9%) was significantly lower than in AQP4-ON (92.3%, *p* = 0.007). The average visual accuracy of patients with MOG-ON was statistically lower than that of AQP4-ON patients (*p* = 0.023).

**Table 1:** The clinical characteristic of enrolled patients and health participants

	MOG-ON (*n* = 17, eyes = 26)	AQP4-ON (*n* = 26, eyes = 40)	HC (*n* = 30, eyes = 60)
Number of patients	17	26	30
Gender (Male / Female)	8/9*	2/24	9/21
Age (years)	34.88 (14.12)	40.50 (11.99)	38.62 (12.20)
ON history (unilateral / bilateral)	8/9	12/14	NA
Number of eyes	26	40	60
ON disease duration (mon, per eye)	42.0 (6–156)	54.0 (8–144)	NA
Number of ON episodes (n, per eye)	1.35 (0.56)	2.05 (1.84)	NA
Duration since last ON (mon, per eye)	12 (6–156)	24 (6–144)	NA
Visual accuracy (logMAR, per eye)	0.33 (0.26)*	0.66 (0.68)	NA
EDSS	3.00 (1–6.5)	3.5 (2–6.5)	NA

*AQP4* Aquaporin4-IgG seropositive, *EDSS* Expanded disability status scale, *HC* Health controls, *MOGAD* MOG-Ab-associated disease, *ON* Optical neuritis, *NA* Not applicable, * *p*＜0.05

### The retinal structure and vessel density in patients with MOGAD-ON

**Fig. 1:**
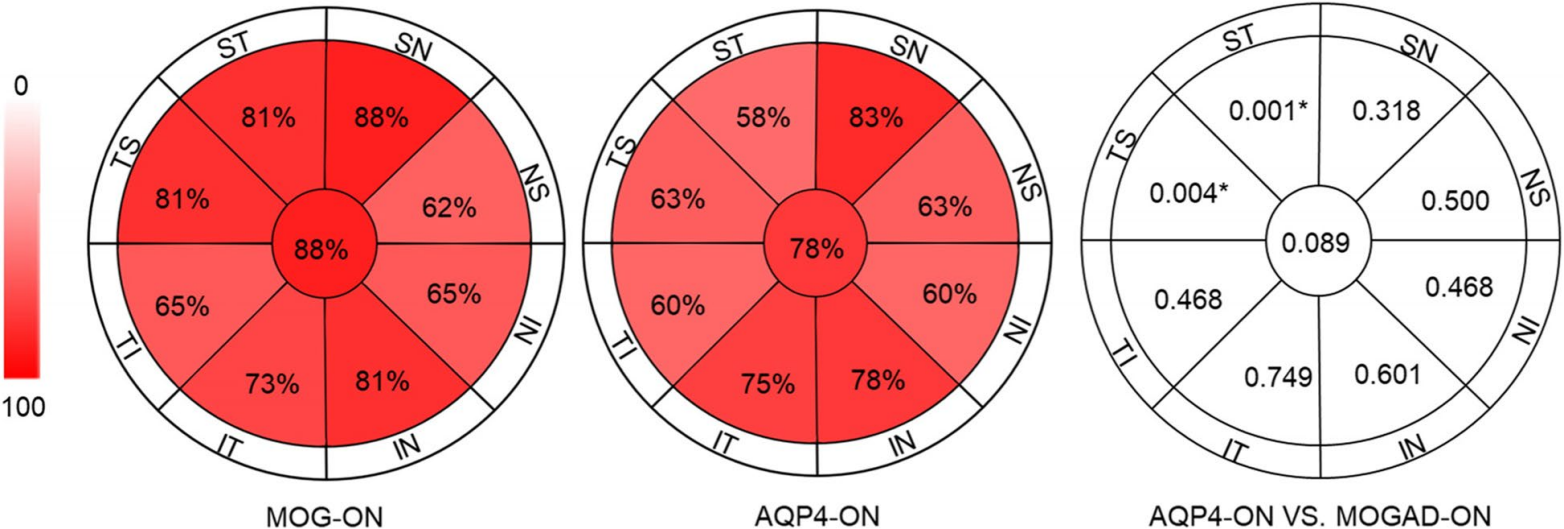
The proportion of eyes with reduced RNFL in each quadrant (MOGAD-ON and AQP4-ON). Compared with the manufacturer’s specification data, MOGAD-ON eyes with reduced thickness of RNFL in different quadrants accounted for 62–88%. AQP4-ON eyes with reduced thickness of RNFL in different quadrants accounted for 58–83%. The proportion of MOG-ON eyes had reduced RNFL thickness in the superior-temporal and temporal-superior regions, which were significantly higher than that of AQP4-ON eyes (58–63%, *p* = 0.004 in temporal-superior area, *p* = 0.001 in superior-temporal area). AQP4-ON: Aquaporin4-IgG seropositive optical neuritis, MOGAD-ON: MOG-Ab-associated disease optical neuritis, TS: temporal-superior quadrant; ST: superior-temporal quadrant; SN: superior-nasal quadrant; NS: nasal-superior quadrant; NS: nasal-superior quadrant; IN: inferior-nasal quadrant; NI: nasal-inferior quadrant; IT: inferior-temporal quadrant; TI: temporal-inferior quadrant

Detailed afferent visual system parameters analyzed by OCT and OCTA were respectively summarized and compared in Table 2 and Table 3. Compared with the manufacturer’s normative data, the thicknesses of RNFL in all sections were reduced in 62–88% of MOGAD eyes (Fig. 1) and significantly lower than HC group (*p* < 0.0001 for all). Besides, the thickness of the inner retina (GCC) was significantly thinner in MOGAD-ON than in HC (*p* < 0.001).

**Table 2:** Retina structural data of MOGAD-ON eyes in comparison to AQP4-Ab-ON eyes and HC

	MOG-ON	AQP4-ON	HC	MOG-ON VS. AQP4-ON			MOG-ON VS. HC		
	Eye = 26	Eye = 40	Eye = 60	B	SE	p	B	SE	p
pRNFL (um)	75.49 (17.98)	78.69 (18.59)	119.74 (10.08)	−0.015	0.016	0.350	−0.238	0.071	0.001*
TEM-RNFL (um)	49.80 (16.90)	55.37 (14.78)	84.03 (9.38)	−0.020	0.019	0.310	−0.163	0.039	0.000*
SUP-RNFL (um)	92.84 (24.56)	96.56 (29.42)	146.31 (14.71)	−0.010	0.010	0.317	−0.305	0.117	0.009*
NAS-RNFL (um)	65.48 (14.25)	64.88 (14.25)	96.03 (15.68)	−0.003	0.019	0.857	−0.134	0.031	0.000*
INF-RNFL (um)	93.01 (28.18)	97.79 (29.26)	152.58 (17.51)	−0.008	0.010	0.392	−0.103	0.025	0.000*
GCC (um)	74.71 (10.19)	74.28 (13.70)	99.12 (5.07)	−0.013	0.022	0.555	−0.029	0.018	0.000*
Outer Retina (um)	172.21 (6.63)	175.67 (8.82)	175.62 (7.55)	−0.058	0.039	0.137	−0.184	0.029	0.062

*AQP4-ON* Aquaporin4-IgG seropositive optical neuritis, *B* Beta, *GCC* Ganglion cell complex, also refer to the inner retina, *HC* Health controls, *INF* Inferior quadrant, *MOGAD-ON* MOG-Ab-associated disease optical neuritis, *NAS* Nasal quadrant, *pRNFL* peri-papillary retinal nerve fiber layer, *SUP* Superior quadrant, *SE* Standard error, *TEM* Temporal quadrant, * p＜0.05

**Table 3:** Retina angiography data of MOGAD-ON eyes in comparison to AQP4-Ab-ON eyes and health controls

	MOG-ON	AQP4-ON	HC	MOG-ON VS. AQP4-ON			MOG-ON VS. HC		
	Eye = 26	Eye = 40	Eye = 60	B	SE	p	B	SE	p
Optic disk RPC (%)	49.53 (4.87)	49.14 (6.42)	55.35 (3.82)	−0.072	0.051	0.154	−0.288	0.069	0.000*
Capillary RPC (%)	52.74 (5.98)	48.63 (7.51)	51.14 (2.13)	−0.058	0.046	0.203	−0.808	0.194	0.000*
Superficial retina VD									
Whole VD (%)	44.22 (4.69)	46.01 (6.31)	51.99 (2.88)	−0.041	0.041	0.320	−0.560	0.122	0.000*
Fovea (%)	13.16 (6.43)	15.31 (6.02)	19.91 (6.60)	−0.045	0.045	0.310	−0.168	0.452	0.000*
para-TEM (%)	46.13 (5.55)	48.27 (6.56)	53.31 (3.38)	−0.029	0.039	0.462	−0.323	0.074	0.000*
para-SUP (%)	47.93 (6.07)	49.26 (6.77)	54.53 (4.02)	−0.039	0.043	0.366	−0.263	0.065	0.000*
para-NAS (%)	44.64 (6.16)	47.93 (6.57)	52.83 (3.86)	−0.090	0.044	0.040*	−0.315	0.073	0.000*
para-INF (%)	45.22 (6.73)	47.87 (6.84)	53.43 (4.76)	−0.066	0.042	0.110	−0.231	0.054	0.000*
peri-TEM (%)	42.54 (3.56)	44.30 (5.56)	48.40 (2.77)	−0.097	0.058	0.094	−0.498	0.103	0.000*
peri-SUP (%)	45.01 (5.18)	46.54 (7.39)	53.09 (2.98)	−0.043	0.428	0.313	−0.511	0.116	0.000*
peri-NAS (%)	46.73 (6.0)1	48.92 (6.83)	55.89 (2.77)	−0.059	0.436	0.173	−0.507	0.112	0.000*
peri-INF (%)	44.41 (6.07)	46.27 (7.34)	52.53 (3.17)	−0.053	0.417	0.207	−0.402	0.091	0.000*
Deep retina VD									
Whole VD (%)	48.01 (7.58)	52.61 (6.72)	54.84 (5.41)	−0.091	0.040	0.022*	−0.163	0.045	0.000*
Fovea (%)	29.28 (7.03)	31.99 (6.88)	37.12 (8.06)	−0.046	0.041	0.259	−0.123	0.036	0.001*
para-TEM (%)	54.38 (8.16)	57.78 (4.84)	59.57 (3.72)	−0.086	0.050	0.087	−0.192	0.062	0.002*
para-SUP (%)	53.75 (7.39)	57.73 (5.19)	58.25 (4.35)	−1.488	1.532	0.044*	−0.137	0.048	0.004*
para-NAS (%)	53.75 (8.18)	57.64 (5.69)	59.47 (4.24)	−0.107	0.049	0.029*	−0.172	0.053	0.001*
para-INF (%)	50.77 (8.44)	56.03 (5.98)	57.38 (5.32)	−0.101	0.042	0.016*	−0.142	0.042	0.001*
peri-TEM (%)	51.52 (6.90)	54.68 (6.76)	57.86 (4.86)	−0.073	0.039	0.062	−0.197	0.052	0.000*
peri-SUP (%)	48.12 (9.65)	53.18 (8.12)	56.40 (6.65)	−0.063	0.032	0.052	−0.129	0.038	0.001*
peri-NAS (%)	47.18 (9.17)	54.19 (7.45)	55.01 (6.50)	−0.098	0.035	0.005*	−0.130	0.037	0.000*
peri-INF (%)	47.58 (8.55)	52.86 (8.09)	55.21 (6.70)	−0.073	0.033	0.028*	−0.125	0.036	0.000*

*AQP4-ON* Aquaporin4-IgG seropositive optical neuritis, *B* Beta, *GCC* Ganglion cell complex, *HC* Health controls, *INF* Inferior quadrant, *MOGAD-ON* MOG-Ab-associated disease optical neuritis, *NAS* Nasal quadrant, *pRNFL* peri-papillary retinal nerve fiber layer, *RPC* radial peripapillary capillaries,  *SUP* Superior quadrant, *SE* Standard error, *TEM* Temporal quadrant, *VD* Vessel densities, * *p*＜0.05

As for the retinal vessel density between MOGAD-ON and HC, the average vessel densities of optic disk radial peripapillary capillaries (RPC) and retina in MOGAD-ON were significantly lower than that in HC (*p* < 0.0001 for all, see Table 3). Moreover, the parafoveal and perifoveal vessel densities in each quadrant were also dramatically reduced in MOGAD-ON compared with the HC group (Table 3).

### The difference in retinal structure and vessel density between MOGAD-ON and AQP4-ON groups

81% of MOG-ON eyes had reduced RNFL thickness in the superior-temporal and temporal-superior regions, which were significantly higher than that of AQP4-ON eyes (58–63%, *p* = 0.004 in temporal-superior area, *p* = 0.001 in superior-temporal area, Fig. 1). The RNFL thickness of each quadrant and the thickness of the inner and outer retinal layers show no significant difference between MOGAD-ON and AQP4-ON eyes (Table 2). Regarding superficial retinal capillary plexus (SRCP), the nasal parafoveal vessel densities were significantly decreased in MOGAD-ON eyes compared to AQP4-ON eyes (B = − 0.090, SE = 0.044, *p* = 0.040, Table 3).

As for the deep retina capillary plexus (DRCP), the whole vessel density (*p* = 0.022), parafoveal vessel densities in the superior, nasal and inferior area (*p* = 0.044, *p* = 0.029, *p* = 0.016, respectively), and perifoveal vessel densities in nasal and inferior area (*p* = 0.005, *p* = 0.028) were significantly decreased in the MOGAD-ON eyes than that in AQP4-ON eyes (Table 3).

### The correlation analysis between OCTA and OCT parameters in ON episodes

GEE analyses were adjusted for age, gender, disease duration, and relapse times of ON, and revealed that the superficial retinal vessel densities of all sectors were positively correlated with the thickness of RNFL and inner retina layer in all patients (*p* < 0.0001 for all, Table 4). For deep retinal vessel densities, parafoveal and perifoveal vessel densities in all sectors were also significantly positively correlated with the thickness of the outer retina layer (*p* < 0.05 for all. Table 4).

**Table 4:** The correlation between OCT angiography parameters and OCT parameters in ON

	RNFL Average			Inner Retina (GCC)			Outer Retina		
	B	SE	p	B	SE	p	B	SE	p
**Superficial retinal capillary plexus**									
Whole VD (%)	2.231	0.321	0.000*	1.568	0.226	0.000*	−0.021	0.208	0.918
Fovea (%)	1.501	0.312	0.000*	0.894	0.229	0.000*	−0.175	0.175	0.317
para-TEM (%)	1.683	0.326	0.000*	1.082	0.231	0.000*	−0.033	0.181	0.856
para-SUP (%)	1.607	0.309	0.000*	0.968	0.231	0.000*	−0.021	0.180	0.907
para-NAS (%)	1.572	0.318	0.000*	0.891	0.239	0.000*	0.067	0.186	0.719
para-INF (%)	1.528	0.295	0.000*	1.204	0.197	0.000*	−0.042	0.173	0.810
peri-TEM (%)	2.360	0.388	0.000*	1.675	0.271	0.000*	−0.049	0.2391	0.839
peri-SUP (%)	1.928	0.257	0.000*	1.239	0.195	0.000*	0.280	0.127	0.027*
peri-NAS (%)	2.111	0.282	0.000*	1.323	0.214	0.000*	0.261	0.128	0.042*
peri-INF (%)	1.687	0.265	0.000*	1.282	0.173	0.000*	0.020	0.166	0.904
**Deep retinal capillary plexus**									
Whole VD (%)	0.226	0.320	0.480	0.093	0.225	0.678	0.462	0.152	0.002*
Fovea (%)	1.140	0.319	0.000*	0.695	0.229	0.002*	−0.008	0.179	0.965
para-TEM (%)	0.347	0.407	0.394	0.053	0.284	0.851	0.458	0.177	0.010*
para-SUP (%)	0.063	0.387	0.871	−0.138	0.267	0.605	0.472	0.166	0.004*
para-NAS (%)	0.242	0.349	0.488	−0.089	0.242	0.713	0.459	0.157	0.003*
para-INF (%)	0.048	0.319	0.881	0.008	0.223	0.970	0.440	0.145	0.002*
peri-TEM (%)	0.422	0.331	0.202	0.230	0.232	0.320	0.380	0.156	0.015*
peri-SUP (%)	0.197	0.564	0.453	0.021	0.185	0.910	0.3765	0.123	0.002*
peri-NAS (%)	0.052	0.265	0.844	−0.037	0.185	0.840	0.369	0.117	0.002*
peri-INF (%)	0.271	0.265	0.306	0.259	0.183	0.157	0.365	0.132	0.006*

*B* Beta, *GCC* Ganglion cell complex, *INF* Inferior quadrant, *NAS* Nasal quadrant, *pRNFL* peri-papillary retinal nerve fiber layer, *SUP* Superior quadrant, *SE* Standard error, *TEM* Temporal quadrant; VD: vessel densities, * *p*＜0.05

### The correlation analysis between visual function and the parameters of OCT and OCTA

In univariate analysis of parameters in MOGAD-ON, the LogMAR was significantly correlated with the duration since last ON (*p* = 0.043), average pRNFL thickness (*p* = 0.037), average inner retina thickness (*p* = 0.034), and the average vessel density of superficial retina (*p* = 0.001). Above factors were selected for multivariate modeling. After adjustment by multivariable linear regression, the LogMAR was significantly correlated with vessel density of SRCP (B = − 0.034, SE = 0.009, *p* = 0.001).

As for AQP4-ON cases, in univariate analysis, the LogMAR was significantly related to the disease duration (*p* = 0.004), average pRNFL thickness (*p* < 0.001), average inner retina thickness (*p* < 0.001), the average vessel density of superficial retina (*p* < 0.001) and average vessel density of deep retina (*p* = 0.005). Above factors were selected for multivariate modeling. The LogMAR was significantly related to the thickness of inner retina layer (B = − 0.043, SE = 0.005, *p* < 0.001), rather than any retinal vessel density.

## Discussion

In this study, we mainly demonstrated differences of retinal microstructure and vasculature between MOGAD-ON and AQP4-ON. MOGAD-ON eyes presented with severe thinning of RNFL, the extent and quadrant of which were comparable to eyes with AQP4-ON. The vessel densities of DRCP in the macular area of MOGAD-ON eyes were significantly lower than that of AQP4-ON eyes. Furthermore, the reduced microvascular densities were positively correlated with the deterioration of the visual acuity in MOGAD-ON.

MOGAD has gradually been a separate disease entity from NMOSD as the distinct pathologies mediated by MOG-Abs. In previous studies, changes in the retinal structure in MOGAD and NMOSD eyes have been separately described [26, 27]. Consistent with the earlier observations of pRNFL thinning and ganglion cell loss in MOGAD, we also described that the average thickness of RNFL in MOGAD-ON eyes was significantly lower than that in normal eyes. It is indicated that the apoptosis of retina ganglion cells and the retrograde degeneration of its axons existed in MOGAD-ON eyes.

However, OCT findings regarding comparing retinal microstructure changes between MOGAD-ON and AQP4-ON eyes have been inconclusive. Some studies have shown that compared with AQP4-Ab-positive NMOSD, MOGAD-ON led to less severe retinal damages [28, 29], but others including us found comparable thinning of the RNFL and GCIPL between these two groups [16, 26]. AQP4-Abs could directly attack AQP4 proteins highly expressed on the surface of Müller cells (mainly in the INL) and astrocytes (mainly in the RNFL) [18]. Müller cells are involved in various homeostatic functions of the retina. The loss of AQP4 reduces the capability of Müller cells to maintain osmotic pressure and induces retinal inflammation, leading to irreversible damage to the retina [30]. Unlike AQP4, MOG is a myelin protein specifically expressed at the outermost surface of myelin sheaths and oligodendrocyte membranes [31], and the autoimmune response to MOG causes demyelination [32]. The retinal ganglion cells are myelinated by oligodendrocytes when the bundle passes through the lamina cribrosa [33]. Consequently, retinal changes observed in MOGAD are expected to be a retrograde degenerative process.

The OCTA data regarding MOG-Ab-associated retinal vessel degeneration are scarce. In conformity with previous study [17], the microvascular densities of SRCP and DRCP in MOGAD eyes were significantly lower than that of normal eyes. We further found that retinal microvascular densities, especially in the DRCP, were markedly lower in MOGAD than in AQP4-Abs-ON. Similar to the previous studies [34], the whole deep retinal vessel densities were also significantly positively correlated with the thickness of the outer retina layer. Retinal microcirculation is high oxygen extraction system whose flow is related with local neuronal activity [35]. The density of the capillary plexus used for oxygen diffusion in the retina is more compressed in the deep layer than surface layer [9], thus any insult to it could lead to retinal vascular abnormalities. The retinal blood vessels share similar anatomic, physiological, and embryological characteristics to the cerebral vessels. It seems to indicate that the presence of MOG-Ab may be related to more severe vascular damage. A case of MOGAD presented with primary CNS vasculitis with perivascular inflammatory cell infiltration [36]. And another study confirmed that all vessels in MOG-Abs-related demyelinating lesions were accompanied by macrophages during the acute phase, which was clearly different from AQP4-Abs-positive NMOSD [37]. Therefore, we speculate that MOG-Abs might cause the retinal destruction and degeneration by leading the peri-microvascular inflammation, rather than directly damaging retinal ganglion cells and their axons.

MOGAD-ON relatively preserved visual acuity compared with AQP4-ON, even if the severity of retinal damages was similar [38]. Unlike AQP4-IgG seropositivity ON, poor visual accuracy was related to changes in retinal structure and function [38], and the deterioration of visual accuracy in MOGAD patients was associated with the reduction of microvascular density of SRCP after adjusting the correlation. Visual stimulation can increase neural activity and cerebral blood flow under normal physiological conditions [39]. Therefore, we speculate that when the retinal microvascular structure is damaged by inflammatory or other pathologies, retinal perfusion becomes insufficient and affects the activity of retinal ganglion cells, and causes photoreceptor damages in MOGAD-ON.

The limitations of our study mainly included that the sample size was relatively small, which is expected given the rarity of these conditions. Whether the vascular changes precede the changes of retinal structure or are secondary to retinopathy cannot be proved. In addition, we didn’t include eyes that were positive for MOG-Ab and AQP4-Ab because “double positive” cases were sporadic.

In conclusion, the retinal neuro-axonal damages in MOGAD-ON were comparable to AQP4-Abs-positive ON. Compared to AQP4-Abs, the MOG-Abs might be related to more severe vascular damage. And the reduced microvascular densities were positively correlated with the deterioration of the visual acuity in patients with MOGAD-ON. Here, we stressed the different pathophysiology between MOGAD-ON and AQP4-ON, but the mechanisms by which MOG-Abs destroys the retinal structure and function are still unclear.

## Data Availability

The datasets used and/or analysed during the current study available from the corresponding author on reasonable request.

## Abbreviations

MOG-Abs: Antibodies against myelin-oligodendrocyte-glycoprotein
MOGAD: Antibodies against myelin-oligodendrocyte-glycoprotein associated disease
AQP4-Ab: Aquaporin-4 antibody
BCVA: Best-Corrected Visual Acuity
CNS: Central nerve systems
CBA: Cell-based assay
DRCP: Deep retinal capillary plexus
ETDRS: Early Treatment Diabetic Retinopathy Study
GCL: Ganglion cell layer
GCC: Ganglion cell complex
HCs: Health controls
INL: Inner nuclear layer
ISL: Inner segment layer
IPL: Inner plexiform layer
IN: Inferonasal
IT: Inferotemporal
INF: Inferior section
MRI: Magnetic resonance imaging
NMOSD: Neuromyelitis optica spectrum disease
NAS: Nasal section
NI: Nasal inferior
NS: Nasal superior
ON: Optic neuritis
OCT: Optical coherence tomography
OCTA: Optical coherence tomography angiography
ONH: Optic nerve head
OPL: Outer plexiform layer
ONL: Outer nuclear layer
OSL: Outer segment layer
pRNFL: Peripapillary retinal nerve fiber layer
RCP: Retina capillary plexus
RPE: Retinal pigment epithelium
RNFL: Retinal nerve fiber layer
RPC: Radial peripapillary capillaries
ST: Superotemporal
SN: Superonasal
SUP: Superior section
SRCP: Superficial retinal capillary plexus
3D: Three-dimension
TI: Temporal inferior
TS: Temporal superior
TEM: Temporal section
VF: Visual field
